# Unsuspecting Dietary Factors in Hyperkalemia: A Case Report on Why History Matters

**DOI:** 10.5811/cpcem.2020.3.43095

**Published:** 2020-04-23

**Authors:** Kevin McLendon, Matthew Wiggins, Derek Hunt, Alex Gauthier, Deepu Thoppil

**Affiliations:** * Merit Health Wesley, Department of Emergency Medicine, Hattiesburg, Mississippi; † William Carey University College of Osteopathic Medicine, Department of Emergency Medicine, Hattiesburg, Mississippi; ‡ Merit Health Wesley, Department of Internal Medicine, Hattiesburg, Mississippi

**Keywords:** Hyperkalemia, diet, history taking, dietary habits, sorbitol and diarrhea

## Abstract

**Introduction:**

We present a case of hyperkalemia secondary to excessive dietary intake of hard caramel candies.

**Case Report:**

An 88-year-old male who presented with acute abdominal pain and vomiting was found to have hyperkalemia of 6.9 milliequivalents per liter. He was stabilized, treated, and discharged the following day after resolution. The cause was identified as his daily consumption of 200 hard caramel candies.

**Discussion:**

The patient had been consuming sugar-free candies, which induced a chronic diarrhea. This led to potassium wasting and augmentation of his home medications. When he transitioned to eating regular caramel candies, he retained too much potassium leading to his presentation.

**Conclusion:**

While often overlooked, dietary history is a crucial part of history-taking to ensure that the underlying cause for illness is discovered and addressed.

## INTRODUCTION

In this case of hyperkalemia a patient with previous, stable, chronic kidney disease (CKD) and regular primary care induced his own hyperkalemia secondary to obscure dietary habits. Dietary habits and nutrition are an often-neglected aspect of medical education, especially for the emergency physician. However, for our patient’s health, it is imperative that we discover the causative factor for his or her presentation to best prevent the condition from reoccurring. This is much more critical in the setting of hyperkalemia as mild elevations have been associated with short- and long-term increased mortality.^1^

Hyperkalemia is often associated with CKD with prevalence as high as 20%. It has been suggested that in CKD above-normal potassium concentrations are present to induce stress on the remaining functional renal cells, which respond to additional diuresis of the potassium.^2^ This delicate system is able to maintain homeostasis in the absence of any additional insult. The insult may come from acute changes in renal excretion, fecal excretion, dietary intake, or metabolic acidosis.^1^ While dietary potassium has been implicated in the past, no previous literature suggests sugar substitutes in food as a causative factor.^2^

## CASE REPORT

An 88-year-old male presented to the emergency department (ED) with a one-day history of nausea and vomiting associated with severe, crampy, and diffuse abdominal pain beginning that morning. The patient also reported daily diarrhea for longer than one month, but it was never problematic and had spontaneously resolved approximately one week prior to arrival. He described his pain as similar to what he had experienced in a previous episode of peritonitis as a young man, which required an exploratory laparotomy; he also reported a history of multiple abdominal surgeries. The vomit was non-bilious and non-bloody; he stated it looked just like the tea he regularly drinks. He denied any associated symptoms of fever, chills, weakness, or body aches.

The patient’s last primary care visit was two weeks prior to arrival where routine labs including renal function and electrolytes were performed. All studies were reported within his baseline. He had an extensive medical history that included diet-controlled type 2 diabetes mellitus, hypertension, hyperlipidemia, coronary artery disease, chronic obstructive pulmonary disease, and stable stage 3A CKD. Baseline renal function was recorded as creatinine 1.60 milligrams per deciliter (mg/dL) (normal range 0.6–1.3 mg/dL) and glomerular filtration rate (GFR) of 45 milliliters per minute per 1.73 meters squared (mL/min/1.73 m^2^) (rnormal range for non-African American ≥ 60).

While he had spontaneous improvement of his abdominal pain prior to emergency physician assessment he continued to have generalized tenderness to palpation, without rebound. We obtained a complete blood count with differential, a comprehensive metabolic panel, and a computed tomography (CT) of the abdomen and pelvis. The initial laboratory results revealed hyperkalemia of 6.9 milliequivalents per liter (normal 3.6–4.9 mmol/L, critical high >6.0 mmol/L), which was not included on the original differential diagnosis. We also noted mild worsening of his baseline renal function with an increase of creatinine to 1.75 mg/dL, and decrease of GFR to 35 mL/min/1.73m^2^. Once the hyperkalemia was identified as a non-hemolyzed sample, therapy was initiated with one-gram intravenous (IV) calcium gluconate, performance of an electrocardiogram (ECG), one liter normal saline fluid bolus, 10 units IV insulin, 25 grams D50, plus 7.5 mg nebulized albuterol. The ECG revealed a paced rhythm at 60 beats per minute and T-wave inversions in leads V3–V6 without hyperacute T waves (Image). The CT revealed no acute process, and the patient was admitted to the hospital for further management and treatment of his hyperkalemia.

Once admitted, the patient reported a long history of xerostomia, which he self-treated by eating hard caramel candies throughout the day for many months. His medication list included clopidogrel, atorvastatin, enalapril, and spironolactone. At this time a lengthy conversation ensued in an attempt to determine the cause of his sudden, symptomatic hyperkalemia. It was determined that he previously had been taking supplemental potassium while he was taking furosemide. In November he discontinued the supplemental potassium because his serum level was “borderline high.” In December, he switched from furosemide to spironolactone due to a recurrence of hypokalemia. His monthly follow-up in January revealed a normal potassium, but he was subsequently admitted two weeks later for this episode of hyperkalemia with abdominal pain (Figure).

**CPC-EM Capsule**

What do we already know about this clinical entity?

Hyperkalemia is a potentially fatal abnormality that is often caused by medications or kidney disease.

What makes this presentation of disease reportable?

Underlying cause in this patient is dietary source which is often an overlooked piece in the patient history.

What is the major learning point?

Do not overlook the diet as a cause, and remember to search for the underlying etiology when treating pathology.

How might this improve emergency medicine practice?

As a reminder to pause and take more inclusive histories from our patients, which can become underrated in the busy ED setting.

His diet consisted primarily of canned soups, sandwiches, and one gallon of sugar-free peach iced tea daily. He further reported consuming a large quantity of hard caramel candies each week. When asked about the candy he stated that he bought a 10-pound bag weekly for himself, roughly 200 candies consumed daily. During his primary care visit in December, he switched to sugar-free caramel candies as part of an attempt at improving his glucose control. Also, at this visit furosemide was discontinued, and he was started on spironolactone (Figure 2). This happened to coincide with the onset of his reported chronic diarrhea. One week prior to ED presentation, he stopped eating the sugar-free candies because the taste “just wasn’t the same,” and switched back to the original caramel candies. This resolved his diarrhea, although he had never noticed the correlation until after his conversation with care providers.

## DISCUSSION

Potassium regulation is dependent on dietary intake and renal and bowel excretion. In a patient without CKD this likely would not have caused the dramatic response seen here. While high dietary potassium can overcome the body’s ability to adapt to excess excretion, the patient was also augmenting his own reserve by the amount of sorbitol-induced diarrhea prior to his cessation from sugar-free candies. Review of diet discovered the patient had an abnormally high daily intake of both 200 caramel candies and one gallon of sugar-free, peach iced tea. From the tea, he was consuming approximately 16 servings of potassium-based sweetener daily. Although no data was available on the true quantity of potassium in each serving, there is literature to suggest potassium-based sweetener substitutes have higher bioavailability than natural potassium and are often under-reported quantities of dietary potassium.^1^ In the caramel candy there is no reported potassium, nor evidence of potassium in the ingredients list; however, the sugar-free version contains sorbitol as the sweetener. Sorbitol is known to induce osmotic diarrhea.^4^

The quantity of intake (200 candies/day) explained his chronic diarrhea from mid-December until one week prior to arrival. Osmotic diarrhea is also associated with increased fecal loss of potassium.^5^ The patient likely would have developed hyperkalemia initially when switched from furosemide to spironolactone as it was coupled with enalapril in the setting of CKD.^6^ The presentation was delayed and offset initially by the co-initiation of sorbitol intake from sugar-free caramel candies, which increased fecal potassium loss and allowed his previous outpatient potassium level to result within normal limits. Once the excess potassium loss was stopped by switching back to original caramel candies (which contain no sorbitol), the diarrhea resolved, and the medication combination and elevated dietary intake of potassium culminated in the patient’s presentation. If untreated, his renal function would likely have deteriorated further and his potassium may have continued to elevate to fatal levels.

CKD places a patient at increased risk for hyperkalemia, especially in the setting of concurrent use with potassium-sparing diuretics.^2^,^6^ As a dietary cause, salt substitutes are a well-known source of potassium.^3^ This case presents an entirely new class of food, sugar-free items, as a causative factor for hyperkalemia. It also includes a unique case of self-temporizing potassium normalization with increased excretion due to the patient’s consumption of sugar-free candies until his abrupt discontinuation in favor of the original versions made with real sugar. The increasing abundance of sugar substitutes in food items may pose a threat to patients in the future. If this patient had not had such high dietary intake of potassium from his potassium-laden, sugar-free tea, he likely would have been found hypokalemic earlier from the sugar-free caramel candy, sorbitol-induced, chronic osmotic diarrhea. Both sugar-free foods in this case can create concerning electrolyte abnormalities on their own and require the attention of the astute provider in history-taking to determine the underlying cause.

## CONCLUSION

This case highlights the importance of good history-taking, as it can mean the difference in diagnosing the underlying cause of a disease and preventing future complications.^7^ More specifically, this case highlights the importance of a dietary history, something too often ignored in the ED. It can be easy to focus on the immediate treatment and stabilization of patients, but we must also be advocates for our patients and remember to search for the underlying cause.

## Figures and Tables

**Figure:**
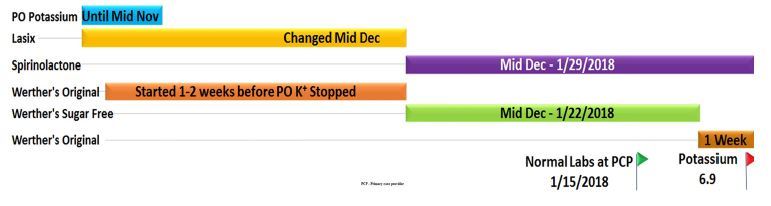
Timeline of medications and caramel candy type in a patient whom developed hyperkalemia.

**Image:**
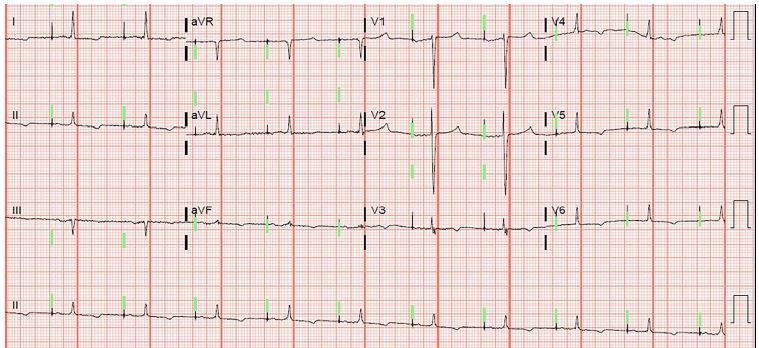
Electrocardiogram of 88-year-old man who presented with sudden, symptomatic hyperkalemia.
